# Radiofrequency Electromagnetic Field Exposure and Apoptosis: A Scoping Review of In Vitro Studies on Mammalian Cells

**DOI:** 10.3390/ijms23042322

**Published:** 2022-02-19

**Authors:** Stefania Romeo, Olga Zeni, Maria Rosaria Scarfì, Loredana Poeta, Maria Brigida Lioi, Anna Sannino

**Affiliations:** 1 CNR, Institute for Electromagnetic Sensing of the Environment (IREA) via Diocleziano 328, 80124 Napoli, Italy; romeo.s@irea.cnr.it (S.R.); scarfi.mr@irea.cnr.it (M.R.S.); poeta.l@irea.cnr.it (L.P.); maria.lioi@unibas.it (M.B.L.); sannino.a@irea.cnr.it (A.S.); 2 Department of Science, University of Basilicata, Viale dell’Ateneo Lucano, 85100 Potenza, Italy

**Keywords:** scoping review, radiofrequency fields, apoptosis, in vitro, quality of studies, qualitative analysis

## Abstract

In the last decades, experimental studies have been carried out to investigate the effects of radiofrequency (RF, 100 kHz–300 GHz) electromagnetic fields (EMF) exposure on the apoptotic process. As evidence-based critical evaluation of RF and apoptosis in vitro is lacking, we performed a scoping literature review with the aim of systematically mapping the research performed in this area and identifying gaps in knowledge. Eligible for inclusion were in vitro studies assessing apoptosis in mammalian cells exposed to RF-EMF, which met basic quality criteria (sham control, at least three independent experiments, appropriate dosimetry analysis and temperature monitoring). We conducted a systematic literature review and charted data in order to overview the main characteristics of included studies. From the 4362 papers retrieved with our search strategy, 121 were pertinent but, among them, only 42 met basic quality criteria. We pooled data with respect to exposure (frequency, exposure level and duration) and biological parameters (cell type, endpoint), and highlighted some qualitative trends with respect to the detection of significant effect of RF-EMF on the apoptotic process. We provided a qualitative picture of the evidence accumulated so far, and highlighted that the quality of experimental methodology still needs to be highly improved.

## 1. Introduction

### 1.1. Rationale

In 2011, the International Agency for Research on Cancer (IARC) classified radiofrequency electromagnetic fields (RF-EMF, 100 kHz–300 GHz) from mobile phones as a possible human carcinogens (2B group) based on the limited evidence from experimental and epidemiological studies [1]. From the literature published since then, and reviewed by international expert panels, the evidence for health effects due to RF-EMF exposure is still inadequate, and needs more accurate investigation [2,3]. Moreover, mechanistic insights of RF-EMF exposure have not been clarified so far for any cellular endpoint. Many hypotheses have been suggested, but none of them has been proven so far [2,3,4,5].

Among the potential cellular mechanisms that are relevant for cancer occurrence, alteration of apoptotic process is of particular interest due to the pivotal role of the regulation of apoptosis in cell homeostasis [6,7] Moreover, abnormalities in cell death regulation, whether they feature insufficient or excessive apoptosis, can be a significant component of other diseases such as autoimmune lymphoproliferative syndrome, AIDS, ischemia and neurodegenerative diseases such as Parkinson’s, Alzheimer’s, Huntington’s diseases and Amyotrophic Lateral Sclerosis [8].

Apoptosis is an important cell death program, highly conserved within multicellular organisms and genetically controlled, which is responsible for the removal of damaged, dysfunctional or no longer necessary cells to promote homeostasis and survival of organisms [6,7,8]. Two pathways are involved in apoptosis that work synergistically to assure the removal of the defective cells. The intrinsic cell death pathway, or mitochondrial pathway, is activated by the cell itself upon detection of cell damage via a number of intracellular sensors. It is governed by the Bcl-2 family of proteins, which regulate commitment to cell death through the mitochondria and the activation of caspase 9. The extrinsic cell death pathway is activated by the interaction between a cell of the immune system and a damaged cell. Activation of the extrinsic cell death pathway occurs following the binding on the cell surface of “death receptors” such as Tumor Necrosis Factor Receptor (Fas TNFR1), or TNF-related apoptosis inducing ligand (TRAIL) receptors, to their corresponding ligands. These death receptors recruit adaptor molecules such as Fas-associated protein with death domain (FADD) and caspase 8. Once the caspases 8 and 9 are activated by inactive pro-caspases, the two pathways converge with the activation of the executioner caspases (caspases 3, 6 and 7). At this point, a cascade of events initiates that leads to DNA fragmentation from activation of endonucleases, destruction of cytoskeleton and nuclear proteins, crosslink of proteins, the expression of ligands for recognition by phagocytic cells, such as the phosphatidylserine, and the formation of apoptotic bodies. The exposure of phosphatidylserine on the external surface of the plasma membrane allows the phagocyte recognition of the dying cells [8,9,10].

Different methods for the detection of apoptosis and its peculiar hallmarks (which allow the recognition with respect to the necrosis), have been developed over time concurrently with the knowledge of apoptosis phenomena. They mainly rely on morphological and biochemical analysis aimed at identifying features of apoptotic cells such as shrinkage, membrane blebbing and chromatin condensation, DNA fragmentation, detection of caspases, cleaved substrates, regulators and inhibitors, externalization of phosphatidylserine, alteration of mitochondrial membrane potential, release of cytochrome-c, analysis of apoptotic or anti-apoptotic regulator proteins such as Bcl-2-associated X protein (Bax), as well as BH3-interacting domain death agonist (Bid), and BCL2 apoptosis regulator (Bcl 2) [8].

A number of in vitro and in vivo experimental studies have addressed the effects of exposures to RF EMF, at frequencies and signals typical of telecommunications, on the apoptotic process. These studies have been carried out under different conditions and experimental regimens with conflicting results, which have not been systematically reviewed. Several reviews regarding the effect of RF-EMF on mammalian cells included apoptosis but were not specifically focused on it [11,12,13,14,15]. Moreover, in all the reviews cited above, papers were not retrieved by performing a systematic literature search, and inclusion criteria did not include cogent quality parameters. The latter have been demonstrated to greatly affect the results of experimental studies. Indeed, quality is emerging as a critical issue in bioelectromagnetic research in general, since the majority of studies do not comply with quality criteria such as adequate attention to dosimetry, inclusion of sham control, positive control, blind evaluation and temperature control [16,17].

As evidence-based critical evaluation of RF exposures and apoptosis is still lacking with reference to health risk assessment, here we performed a scoping literature review, with the aim of systematically mapping the research performed in this area and identifying gaps in knowledge. We focus on in vitro studies because they can provide essential information about the potential effects of chemicals or physical agents on specific cell properties, and allow a more rapid, cost effective and well-controlled approach to molecular and mechanistic studies than conventional laboratory animal models [16]. Moreover, the preamble to the IARC Monographs on the Identification of Carcinogenic Hazards to Humans has given new emphasis and highlighted the importance of mechanistic studies in corroborating evidence and providing biological plausibility to other types of studies, and the possibility that they could provide strong evidence in case of consistent findings demonstrated across a number of different systems and in different species [1].

### 1.2. Objective

The aim of this scoping review is to survey the available evidence on the effects of RF-EMF exposures on the apoptotic process in mammalian cells cultured in vitro by mapping how research was conducted, by identifying key characteristics of the experiments and any existing gaps in knowledge. A systematic literature search was performed and the review was restricted to studies that adhere to basic quality criteria defined a priori, and thus characterized by low risk of bias. The scientific question, formulated as a PECO (Population, Exposure, Comparator, Outcome) statement, is outlined in Table 1.

## 2. Methods

The scoping review conforms to PRISMA-ScR (Preferred Reporting Items for Systematic reviews and Meta-Analyses extension for Scoping Reviews) guidelines, provided as Supplementary Material (Table S1: PRISMA-ScR-Checklist) [18,19].

### 2.1. Eligibility Criteria

We have restricted inclusion to peer-reviewed journal articles reporting findings from primary studies and published in English. Meeting abstracts, conference proceedings, and commentaries were excluded, whereas reviews have been used to check for missing articles.

We have included in vitro studies assessing the capability of RF-EMF in the frequency range between 100 kHz and 300 GHz, to affect apoptosis process in mammalian cells (Table 1), with no restrictions on biological model (freshly collected or immortalized cells), cell status (healthy or cancerous), or cell lineage. Regarding the apoptosis outcome, we have included the endpoints reported in the PECO statement (Table 1).

For studies that evaluate apoptosis in relation to both RF exposure alone, and to co-exposure to RF fields and other agents, only findings concerning RF exposure alone have been considered, because we want to focus on potential apoptosis effects of RF-EMF themselves.

In order to restrict the analysis to papers characterized by a low risk of bias, we have adopted the following quality-based exclusion criteria [20].

First, we excluded studies that did not provide information to adequately characterize exposure conditions, such as frequency range, signal type, exposure level and duration, as detailed in the PECO statement (Table 1). We excluded studies where dosimetry analysis was not performed at all or was not carried out with adequate methods. For example, estimation of SAR from measurements of the electric field in absence of the sample is not appropriate because the sample significantly perturbs the electric field in the RF range. On the contrary, estimation of SAR from computation of electric field in the sample or by calorimetric measurements are acceptable [20,21].

We included studies that used sham-exposed controls, i.e., a sample placed in an exposure system identical to that used to administer the treatment, except for the emission of RF-EMF [20]. A further quality-based exclusion criteria was the absence of temperature control. As a main confounding factor for RF exposure, temperature inside the samples must be monitored to ascertain absence of heating, or to counteract possible thermal increase. Finally, we also excluded studies that performed less than three independent experiments or did not report the number of independent experiments.

### 2.2. Information Sources and Search Strategy

Our primary information sources were NCBI PubMed and Web of Science (WOS) databases. In both cases, we did not apply restrictions in terms of time coverage, and the most recent search was performed on 12 August 2021. The search strategies developed for both databases are provided as Supplementary Material (Table S2: Search queries). We also checked reference lists of review papers and authors’ personal literature databases to retrieve studies that were missed by the web search.

### 2.3. Selection of Sources of Evidence

All bibliographic records have been imported into the reference management software Endnote^®^ X9, and the appropriate functions have been used to remove duplicates and classify the papers by relevance and inclusion/exclusion status. Two independent reviewers (SR and MRS) have performed a two-phase selection process: first, the papers have been included/excluded for relevance based on the screening of title and abstract; second, the full text of all potentially relevant papers has been retrieved and assessed for compliance with the predefined eligibility criteria. The results of the papers selection process have been graphically displayed in a PRISMA flow-chart, and the papers excluded at the stage of full-text examination have been recorded in a separate table, with indication of at least one motivation for exclusion.

### 2.4. Data Charting Process and Data Items

The same investigators in charge of the papers selection have also extracted the relevant information regarding the experiments, using the forms reported as Supplementary Material 3. More specifically, for each paper we have identified individual experiments, characterized by different exposure conditions (in terms of frequency, signal, exposure level or duration), or different cell models or endpoints. For each experiment, the following data have been extracted and recorded in the form:

- Complete paper reference.
- Cell type and number of independent experiments.
- Apoptosis endpoint.
- Exposure conditions: frequency, type of signal, exposure metric, exposure duration.
- Results: statistically significant effect (based on the analysis performed by the authors of the study) irrespective of the direction (increase or decrease); non statistically significant effect.
- Comment: any other information useful to further assess the quality of study (e.g., blind analysis, presence of positive control, appropriateness of statistical analysis, etc.)

### 2.5. Synthesis of Results

- We performed descriptive statistics of the selected parameters in order to characterize the experiments over the publication time by cell type (human vs. animal, primary cells vs. cell lines), with respect to the endpoints analyzed, and the exposure conditions. To the latter aim, we identified several subgroups within the exposure parameters:
- Frequency subgroups: 100 kHz to <10 MHz (F1); 10 MHz to ≤6 GHz (F2); >6 to ≤300 GHz (F3);
- Exposure duration subgroups: ≤ 1 h (ED1, acute); >1 h to ≤24 h (ED2, long); >24 h (ED3, chronic, including intermittent exposure over several days);
- Exposure level subgroups: SAR ≤ 1 W/kg or S_ab_ < 20 W/m^2^ or S_inc_ < 10 W/m^2^ (EL1); 1 W/kg <SAR≤ 2 W/kg S_ab_ = 20 W/m^2^ or S_inc_ = 10 W/m^2^ (EL2); SAR> 2 W/kg or S_ab_>20 W/m^2^ or S_inc_>10 W/m^2^ (EL3).

We also assessed the overall incidence of statistically significant or non-significant effects in the experiments, and the relative incidence of effects with respect to the endpoints and the exposure parameter subgroups.

## 3. Results

### 3.1. Selection of Sources of Evidence

**Figure 1:**
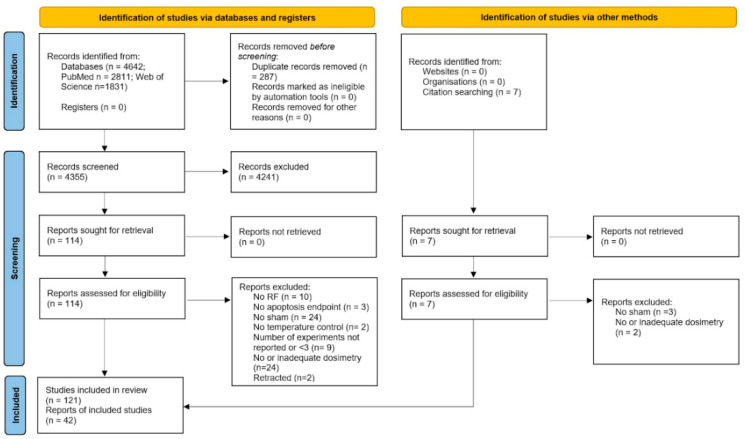
PRISMA flowchart.

The results of the literature search and of the screening process are summarized in the PRISMA flow-chart in Figure 1. The literature search yielded a total of 4649 records, which reduced to 4362 after duplicates removal in Endnote X9. The first round of screening, based on information and terms in the title and abstract, led to the exclusion of 4241 publications, whereas for the remaining 121 records the full text was assessed for eligibility. Among these, 79 papers were excluded because they were not compliant with either basic or quality criteria, whereas the remaining 42 were fully analyzed for data extraction and synthesis. The full references of excluded papers with motivations for exclusion are reported in Table 2.

**Figure 2:**
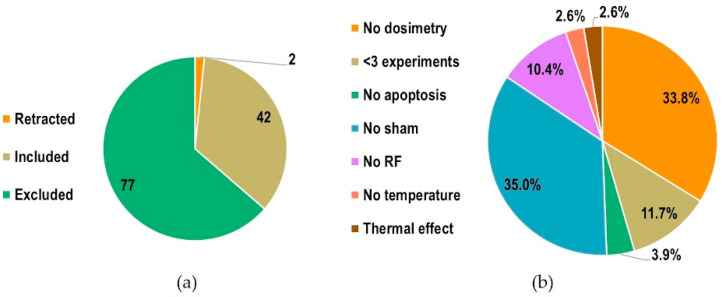
(**a**) Number of included, excluded and retracted studies; (**b**) motivations for exclusion with relative proportions.

Figure 2 shows the number of included, excluded and retracted studies, and the motivations for exclusion with relative proportions (when more than one motivation applied, only one of them was counted): the most recurrent motivations were the absence of sham control and the absence of dosimetry or of appropriate dosimetry methods.

### 3.2. Characteristics of Sources of Evidence

**Figure 3:**
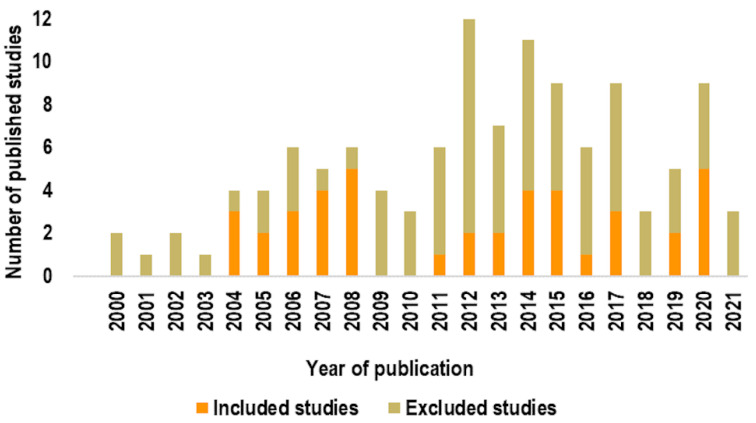
Temporal trend of publication of studies.

Figure 3 reports the temporal trend of publication of the included and excluded studies. The first studies were published in 2000 (2004, if we consider relevant studies that were included based on our quality criteria), meaning that this topic has been addressed in the literature for a relatively short time.

The main characteristics of included studies are charted in Table 3 and Table 4. We have separated studies into two categories. First, those that did not observe statistically significant (according to the statistical analysis performed by the authors) alterations of the apoptotic process due to RF-EMF exposures in any of the experimental conditions considered (Table 3). Secondly, those that did report significant alterations in at least one of the experimental conditions considered (Table 4).

A total of 255 experiments were extracted from the 42 papers analyzed. Each experiment was identified on the basis of either one of the exposure parameters (frequency, signal, exposure level or duration), or of the cell type, or of the endpoint analyzed. Data extracted from each experiment are provided as Supplementary Material (Table S3: Data extracted from experiments).

### 3.3. Results and Critical Appraisal of the Source of Evidence

We surveyed the data extracted from included studies with respect to relevant parameters, namely the cell origin (human vs. animal, primary vs. cell lines), the endpoints analyzed, the frequency, exposure level and exposure duration subgroups, the reporting of statistically significant effects.

**Figure 4:**
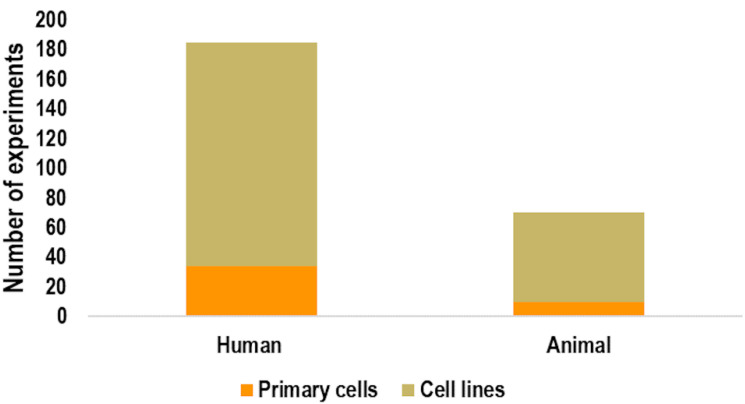
Number of experiments using animal or human cells. For each group, the number of experiments using primary cells vs. cell lines is also reported.

**Figure 5:**
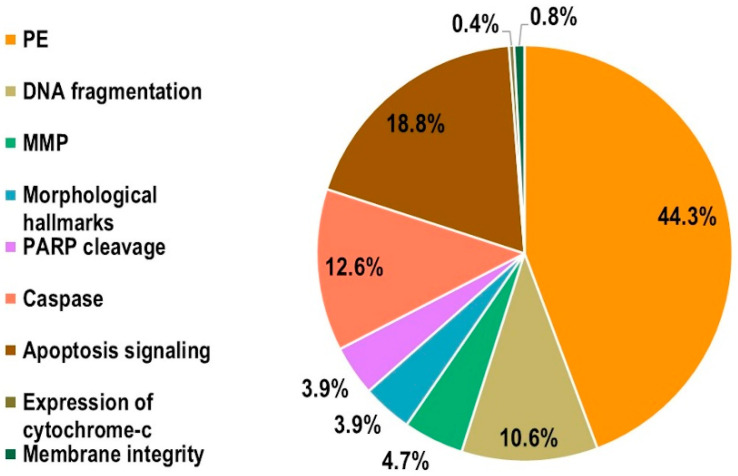
Percentage of endpoints analyzed in the experiments (MMP: mitochondrial membrane potential; PE: phosphatidylserine externalization; PARP: Poly(ADP-Ribose) Polymerase).

As shown in Figure 4, human cells were used as biological model more than animal cells (72.5% vs. 27.5%), whereas in both cases immortalized cell lines (81.6% human and 85.7% animal) were mainly used with respect to primary cells (18.4% human, 14.3% animal). The percentage of endpoints analyzed were as reported in Figure 5, where the vast majority (44.3%) of experiments assessed apoptosis by analyzing the phosphatidylserine externalization, followed by apoptosis signaling (18.8%), caspase activity (12.6%), and DNA fragmentation (10.6%). The remaining 13.7% accounted for the other considered apoptosis endpoints, namely alteration of mitochondrial membrane potential (4.7%), morphological hallmarks (3.9%), PARP cleavage (3.9%), membrane integrity (0.8%), and expression of cytochrome-c (0.4%).

**Figure 6:**
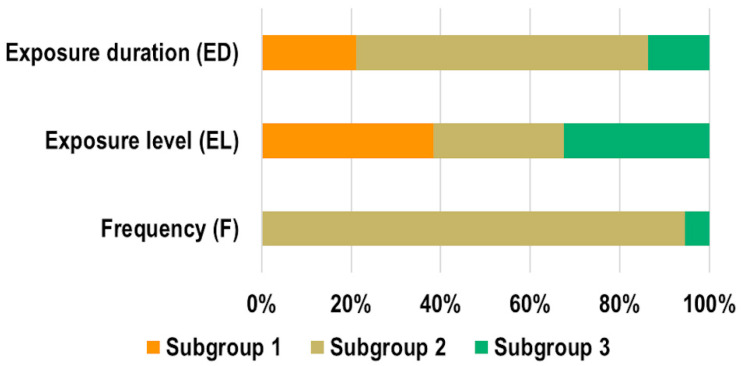
Percentage of experiments belonging to the three subgroups (ED1/ED2/ED3; EL1/EL2/EL3; F1/F2/F3) within the main exposure parameters.

Figure 6 shows the percentage of experiments belonging to the three subgroups (as defined in Table 1) within the main exposure parameters (frequency, exposure level and exposure duration). The majority of the experiments (94.5%) were performed by applying EMF in the F2 subgroup (10 MHz to ≤6 GHz), only 5.5% of them applied EMF in the F3 (>6 to ≤300 GHz) subgroup, and none of them employed frequencies below 10 MHz (F1). The distribution within the exposure level subgroups was definitely more uniform, with 38.4% of experiments performed at SAR < 1 W/kg or S_ab_ < 20 W/m^2^ or S_inc_ < 10 W/m^2^ (EL1), 29% at 1 W/kg < SAR ≤ 2 W/kg S_ab_ = 20 W/m^2^ or S_inc_ = 10 W/m^2^ (EL2), and 32.5% SAR > 2 W/kg or S_ab_>20 W/m^2^ or S_inc_>10 W/m^2^ (EL3). In most of the experiments (65.1%) exposure duration was long (ED2, >1 h to ≤24 h), whereas in 21.2% and 13.7% they were acute (ED1, ≤1 h) and chronic (ED3, >24 h), respectively.

**Figure 7:**
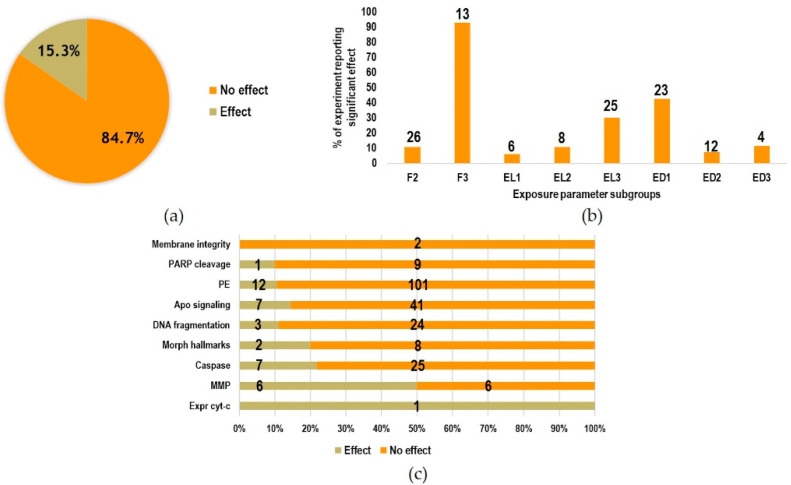
(**a**) Percentage of experiments reporting statistically significant effects and of experiments not reporting effects; (**b**) relative incidence of significant effects in the exposure parameter subgroups (F1 is not reported because no experiments were performed at frequencies below 10 MHz; F2/F3; ED1/ED2/ED3; EL1/EL2/EL3 as defined in Table 1; Number above each bar refer to the absolute number of experiments where significant effects were observed); (**c**) percentage of experiments reporting statistically significant effect or no effect with respect to the endpoints analyzed (numbers on the bars indicate the absolute numbers of experiments performed).

In the majority of experiments (84.7%), no statistically significant effects on the analyzed endpoints were found; only the 15.3% reported statistically significant effects (Figure 7a). The percentage of experiments reporting effects is presented in Figure 7b with respect to exposure parameters. The highest incidence occurred in the F3 subgroup, with 13 out of 14 experiments (belonging to two different studies) reporting significant effects. Regarding the exposure level and duration subgroups, the highest incidence was obtained for above limits (EL3, 30.1%) and acute exposures (ED1, 42.6%). The incidence of significant and non-significant effects with respect to the analyzed endpoints is shown in Figure 7c. The highest incidence of significant effects was found in terms of MMP modifications (6 out of 12 experiments), followed by caspases activation (7 out of 32 experiments) and the observation of morphological hallmarks (2 out of 10 experiments). For the PE, apoptosis signaling and DNA fragmentation endpoints, the incidence of significant effects ranged from 10.6 to 14.3% (12 out of 113 experiments for PE; 7 out of 48 for apoptosis signaling; 3 out of 27 experiments for DNA fragmentation). Only two experiments assessed the membrane integrity endpoint and did not find significant effects. Only one experiment assessed expression of cytochrome-c and found a significant alteration.

Moreover, the incidence of statistically significant effects in experiments performed with human or animal cells was 13.5% and 20%, respectively.

Table 3 reports an overview of the studies that did not observe significant alterations of the apoptotic process due to RF-EMF exposures. In 17 out of 31 studies, only one apoptosis endpoint was analyzed; in 20 out of 31 studies the analysis was not performed in blind; and in 7 out of 31 studies positive control was not included. The experiments in which significant alterations of apoptosis endpoints were found belonged to twelve studies, which are overviewed in Table 4. In 5 out of 11 studies, only one apoptosis endpoint was investigated; in 7 out of 11 studies the analysis was not performed in blind; and in 10 out of 11 studies, positive control was lacking.

## 4. Discussion

### 4.1. Summary of Evidence

One of the main concerns regarding health effects of RF-EMF is that prolonged exposures to weak field levels may cause long-term effects. In spite of the high number of studies published on this matter, the evidence accumulated so far is inconclusive and controversial.

The majority of studies regarding biological effects of RF-EMF are based on an in vitro study design, because this approach is rapid, cost effective, allows exposures to be performed under strictly controlled electromagnetic and environmental conditions and provides insight into mechanistic interactions [1]. Among the biological outcomes that can be of interest for the mechanistic assessment of long-term effects, apoptosis has been increasingly considered over the last twenty years. The gained information has not been comprehensively reviewed and does not allow for a complete picture of investigation carried out so far, of the possible effects on this critical cellular process and of the existing gaps in knowledge.

Manna and Gosh reviewed the effects of RF-EMF exposure in cultured mammalian cells on several biological outcomes, including apoptosis. The authors concluded that RF-EMF exposure might affect the apoptotic process in vitro, with results depending on the type of modulation, intermittent mode of exposure and cell model [15]. Halgamuge and co-workers performed an extensive meta-analysis of data from in vitro studies published between 1990 and 2015, and investigating effects of weak RF-EMF from mobile phones. The analysis revealed a lack of uniform responses in any of the investigated outcomes, which included but was not specifically focused on apoptosis, and also highlighted some recurrent patterns of evidence which depended on cell and signal types [14]. Moreover, in the abovementioned reviews, the study inclusion criteria did not take into account the aspects of quality of experimental methods, which have been widely demonstrated to affect the results [16,17]. In [16], co-authored by two authors of this review, apoptosis was considered together with proliferation to detect possible statistical associations between RF-EMF exposures and cellular response. Cellular response after exposure to RF-EMF was significantly associated to cell lines rather than to primary cells, but not to other experimental parameters.

In this scoping review, for the first time in our knowledge, we specifically addressed apoptosis outcome in studies evaluating the effects of RF-EMF exposures on mammalian cells in vitro. We conducted a systematic literature review, included studies on the basis of quality criteria defined a priori, and provided an overall picture of what has been published so far.

The systematic literature search yielded a total of 121 relevant papers, but only 34.5% of them met the inclusion criteria. The majority of retrieved papers failed to comply with quality criteria for good bioelectromagnetic experiments [16,17,143]. The main motivations for exclusion were the absence of sham controls and the lack of dosimetry analysis, or of appropriate methods for dosimetry analysis. Lack of compliance with these two criteria indicates that experimental conditions were not identical across study groups, and that there was a low confidence in exposure characterization. Quality of experimental methods has become an issue in bioelectromagnetic research, with the majority of published papers presenting flaws on either electromagnetic or biological requirements, or both. Basic quality criteria for in vitro experiments on RF-EMF exposures include the presence of sham control, dosimetry analysis conducted with standardized methods, temperature control, blind analysis and positive control. The absence of some or all of these requirements has been shown to be highly associated with the detection of effect [16,17]. In this review, we considered three out of five basic requirements as inclusion criteria (sham, dosimetry analysis, temperature control). The motivation of this choice was to include papers with a low risk of bias, but we did not consider the absence of blind analysis and of positive control among the exclusion criteria in order to be more inclusive towards the final analysis of relevant papers. The latter criteria were applied for a deeper characterization of the quality of studies. Indeed, in more than half of the included studies analysis of data was not blinded, and in almost half of the studies positive control was not included in the study design.

It is important mentioning that in more than half of the included studies apoptosis was evaluated by assaying a single endpoint. Since apoptosis occurs via a complex signaling cascade that is tightly regulated at multiple points, and since it presents many features in common with necrosis, it is crucial to perform two or more distinct assays, based on different principles, to confirm that cell death occurred via apoptosis [8]. As an example, the detection of phosphatidylserine externalization requires the use of specific dyes (Annexin-V binds to phosphatidylserine on the plasma membrane, while Propidium Iodide only enters necrotic cells) allowing discrimination of apoptotic from necrotic cells, because an increase in membrane permeability is also a feature of necrotic cells. For the same reason, each test must be associated with others based on different apoptotic features.

We have overviewed the main characteristics and outcome of the included studies. The majority of them did not find significant alterations of the apoptotic process due to RF-EMF exposure. Looking at the experiments extracted from the studies, when a statistically significant effect was observed it mainly occurred at frequencies above 6 GHz, and for acute (≤ 1h) exposure durations. However, since the number of studies reporting effects is very small, and the considered experimental conditions are highly heterogeneous, further investigations are needed, together with replication studies, to confirm or confute these results. Moreover, even though the included studies met the basic quality criteria, most of them still presented flaws in the experimental methods (lack of blind analysis and/or positive control, assessment of single endpoints). It can be stated that, to be of value, future studies that investigate the effect of RF-EMF in mammalian cells should aim to be of high methodological quality and be sufficiently powered by performing an adequate number of experiments.

### 4.2. Limitations

The bibliographic search was conducted only on two databases (PubMed and WOS). Even though these two databases may contain the vast majority of studies within the field, it is possible that potentially relevant studies might not be indexed there. Misclassification of studies based on keywords, title or abstract might have also affected the sensitivity of the search strategies. We conducted hand searches to minimize the number of articles missed, but this still may not have captured all eligible articles.

## 5. Conclusions

This scoping review sought to systematically map the research regarding the effects of RF-EMF on apoptosis in mammalian cells, and to identify any existing gaps in knowledge within health risk assessment of RF-EMF exposures. This will definitely facilitate to gain reliable information on the effects of RF exposure on the apoptotic process when in a next step, a quantitative analysis of the papers included in this scoping review will be carried out by mainly addressing questions on the direction of the effect (induction or suppression of apoptosis), effect size, possible dose–response relationship, possible association of the effect size with the quality score of the experiments, and possible major capability of certain exposure parameter ranges to exert an effect. The major gap in knowledge from the qualitative analysis conducted here is the lack of a systematic approach based on quality of the experimental methodologies adopted in the studies retrieved and analyzed in this scoping review.

Therefore, the evidence here presented is a further confirmation that, in spite of the large amount of relevant papers available in the literature, a huge effort still needs to be made in bioelectromagnetic research towards the improvement of experimental quality, which is crucial to guarantee the reliability, robustness and reproducibility of results.

## Figures and Tables

**Table 1:** PECO statement.

**P**opulation	In Vitro Models of Healthy or Cancerous Mammalian Cells, either Immortalized or Freshly Collected via Drawing/Explant.
**E**xposure	Controlled in vitro exposure to radiofrequency radiation (100 kHz-300 GHz), based on suitable exposure metrics. Exposure details: Frequency bands: 100 kHz to <10 MHz; 10 MHz to ≤6 GHz; >6 to ≤300 GHz; Metrics: induced electric field (Eind, V/m) in the 100 kHz-10 MHz range, Specific Absorption Rate (SAR, W/kg) in the 10 MHz–6 GHz range, incident (S_inc_) or absorbed (S_ab_) power density (W/m^2^) in the 6 GHz–300 GHz range; Signal characteristics: continuous waves (CW); pulsed (PW); Duration: ≤ 1 h (acute); >1 h to ≤24 h (long); >24 h (chronic)
**C**omparator	Sham-exposed (sham) control samples.
**O**utcome	Apoptosis assessed by considering the following endpoints: morphological hallmarks (cell shrinkage, plasma membrane blebbing, chromatin condensation, etc.), alteration of mitochondrial membrane potential, cytochrome-c release, translocation of phosphatidylserine, caspases activation, PARP-cleavage, DNA fragmentation, apoptosis signaling (Bak, Bcl-10, Bcl-2, p-53, Bax, Bid, Bag, apoptosis-inducing factor (AIF), etc.).

**Table 2:** Excluded papers with motivations.

ID	Reference	Motivation(s) for Exclusion
1	Alessio et al. 2019 [22]	No dosimetry
2	Al-Serori et al. 2017 [23]	Less than three independent experiments.
3	Asano et al., 2020 [24]	No apoptosis endpoint
4	Asano et al., 2017 [25]	No sham control. Inadequate description of the RF exposure system and dosimetry.
5	Atasoy et al., 2009 [26]	No sham control
6	Avendano et al., 2012 [27]	No sham control
7	Azma et al., 2018 [28]	No dosimetry
8	Ballardin et al., 2011 [29]	No sham control
9	Calabrò et al., 2012 [30]	No dosimetry
10	Cao et al., 2009 [31]	No sham control
11	Caraglia et al., 2005 [32]	No sham control
12	Çiğ and Naziroğlu, 2015 [33]	No sham control
13	Eghlidospour et al., 2017 [34]	No dosimetry
14	Esmekaya et al., 2013 [35]	Number of independent experiments not reported
15	Esmekaya et al., 2017 [36]	No temperature control
16	Falzone et al., 2010 [37]	No sham control
17	Glazer et al., 2010 [38]	Thermal effect
18	Grasso et al., 2020 [39]	No dosimetry
19	Harvey and French, 2000 [40]	Less than three independent experiments
20	Hirose et al., 2006 [41]	Less than three independent experiments
21	Jin et al., 2012 [42]	Less than three independent experiments
22	Jin et al., 2021 [43]	No sham control
23	Jooyan et al., 2019 [44]	No sham control
24	Kahya et al., 2014 [45]	No sham control
25	Karaca et al., 2012 [46]	No sham control
26	Karkabounas et al., 2006 [47]	No dosimetry
27	Kayhan et al., 2016 [48]	No dosimetry
28	Keczan et al., 2016 [49]	Not RF range
29	Kim et al., 2021 [50]	No sham control
30	Korraah et al., 2012 [51]	Not RF range
31	Koshkina et al., 2014 [52]	Thermal effect
32	Lantow et al., 2006 [53]	Not apoptosis
33	Lee et al., 2016 [54]	Number of independent experiments not reported
34	Lee et al., 2005 [55]	Number of independent experiments not reported
35	Lee et al., 2014 [56]	No sham control
36	Leszczynski et al., 2002 [57]	No apoptosis endpoint
37	Li et al., 2014 [58]	No dosimetry
38	Li et al. 2010 [59]	Only combined exposures
39	Li et al. 2011 [60]	Not RF range
40	Li et al. 2012 [61]	No dosimetry
41	Liang et al., 2013 [62]	No dosimetry
42	Liu et al., 2011 [63]	Inadequate description of the RF exposure system and dosimetry.
43	Liu et al., 2012 [64]	No sham control
44	Liu et al., 2015 [65]	No sham control
45	Lu et al., 2012 [66]	No sham control.
46	Maioli et al., 2013 [67]	No sham control. No dosimetry
47	Marinelli et al., 2004 [68]	Absence of appropriate dosimetry methods
48	Martin et al., 2009 [69]	Absence of appropriate exposure metrics and dosimetry
49	Mortazavi et al., 2015 [70]	Absence of appropriate exposure metrics and dosimetry
50	Naziroglu et al., 2015 [71]	No sham control
51	Narvaez et al., 2018 [72]	Absence of appropriate exposure metrics and dosimetry
52	Nishioka et al., 2020 [73]	Absence of appropriate exposure metrics and dosimetry
53	Oh et al., 2001 [74]	Not RF range
54	Ozgur et al., 2014 [75]	Number of independent experiments not reported
55	Ozsobaci et al., 2020 [76]	Absence of appropriate exposure metrics and dosimetry
56	Pacini et al., 2002 [77]	No dosimetry
57	Pastacı Özsobacı et al., 2018 [78]	No sham control. Number of experiments not reported
58	Peinnequin et al., 2000 [79]	No dosimetry
59	Port et al., 2003 [80]	No sham control
60	Radeva et al., 2009 [81]	Not RF range
61	Solek et al., 2017 [82]	Not RF range
62	Song et al., 2011 [83]	No sham control
63	Sueiro-Benavides et al., 2021 [84]	No sham control
64	Tomruk et al., 2019 [85]	Inadequate description of dosimetry. Sham exposures was likely, not concurrent to RF exposure
65	Urnukhsaikhan et al., 2016 [86]	Not RF range
66	Volkova et al., 2014 [87]	No dosimetry
67	Wu et al., 2011 [88]	No sham control
68	Wu et al., 2012 [89]	No dosimetry
69	Wu et al. 2012 [90]	Retracted
70	Xing et al., 2016 [91]	No dosimetry
71	Yang et al., 2012 [92]	No sham control
72	Yao et al. [93]	Retracted
73	Zhang et al., 2013 [94]	Inadequate description of the RF exposure system and dosimetry.
74	Zhao et al., 2007 [95]	Inadequate description of the RF exposure system and dosimetry.
75	Zhao et al., 2017 [96]	No information on dosimetry
76	Zhijian et al., 2013 [97]	Less than three independent experiments
77	Zhou et al., 2008 [98]	No sham control.
78	Zhu et al., 2014 [99]	No sham control. No dosimetry performed.
79	Zuo et al., 2015 [100]	No temperature control at 18 W/kg SAR.

**Table 3:** Overview of studies that did not report statistically significant alterations of the apoptotic process following RF-EMF exposures.

ID	Reference	Cell Type	Biological Endpoint	Exposure Conditions	Results	Comment
1	Belyaev et al., 2005 [101]	Human blood lymphocytes	DNA fragmentation Morphological hallmarks	915 MHz (GSM) 0.037 W/kg 2 h	No effect	-
2	Bourthomieu et al., 2013 [102]	Primary human amniotic cells	Apoptosis signaling	900 MHz (GSM) 0.25, 1, 2, 4 W/kg 24 h	No effect	Non-blinded analysis
3	Capri et al., 2004a [103]	Human blood mononuclear cells	PE MMP modification	1800 MHz (GSM-basic, GSM-talk, DTX) 1.4 and 2 W/kg 44 h (10 min on/20 min off cycles)	No effect	-
4	Capri et al., 2004b [104]	Human blood mononuclear cells	PE MMP modification	900 MHz (CW and GSM) 0.07 and 0.076 W/kg 1 h/day for 3 days	No effect	Non-blinded analysis
5	Chauhan et al., 2007 [105]	Human-derived immune cell lines (HL-60, Mono-Mac-6, TK6)	DNA fragmentation	1900 MHz (PM) 1 and 10 W/kg 6 h (5 min on/10 min off cycles)	No effect	-
6	Chen et al., 2014 [106]	Embryonic mouse neural stem (eNSCs) cells	DNA fragmentation Caspase activity Apoptosis signaling	1800 MHz 4 W/kg 3 days (5 min on/10 min off cycles)	No effect	-
7	Choi et al., 2020 [107]	Human adipose tissue-derived stem (ASCs) cells, liver cancer stem cells (Huh7)	PARP cleavage	1700 MHz (LTE) 1 and 2 W/kg 72 h	No effect	Non-blinded analysis
8	De Amicis et al., 2015 [108]	Human primary fibroblasts HFFF2	PARP cleavage	120 THz (PW) 4 W/m^2^ (0.015–0.022 W/g) 20 min	No effect	Non-blinded analysis No positive control
9	Durdik et al., 2019 [109]	Umbilical cord blood (UCB) cells	PE	900 MHz (GSM), 1950 MHz (UMTS) 4 and 40 W/kg 2 h	No effect	Non-blinded analysis
10	Glaser et al., 2016 [110]	Hematopoietic stem cells (HSC); promyelocytic leukemia cell line (HL-60)	PE	900 MHz (GSM), 1950 MHz (UMTS), 2535 MHz (LTE) 0.5, 1, 2 and 4 W/kg 4 and 20 h (HSC) 4 and 66 h (HL-60)	No effect	Sham and RF samples were not run concurrently
11	Gulati et al., 2020 [111]	Human peripheral blood lymphocytes	PE	923, 1947.47, 1977 MHz (UMTS) 0.04 W/kg 1 h and 3 h	No effect	Non-blinded analysis No positive control
12	Gurisik et al., 2006 [112]	Promyelocytic leukemia cell line (HL-60) Human neuroblastoma cells (SK-N-SH)	Membrane integrity	900 MHz (GSM) 0.2 W/kg 2 h	No effect	Non-blinded analysis No positive control
13	Hook et al., 2004 [113]	Lymphoblastoid Molt-4 cells	PE	812.56 MHz (iDEN) 24 W/kg 836.55 MHz (TDMA) 26 W/kg; 847.74 MHz (CDMA) 835.62 MHz (FDMA) 3.2 W/kg 2, 3, 21 h	No effect	Non-blinded analysis
14	Hoyto et al., 2008a [114]	Human neuroblastoma (SH-SY5Y); Mouse fibroblasts (L929)	Caspase activity DNA fragmentation	872 MHz (CW and GSM) 5 W/kg 24 h	No effect	Non-blinded analysis
15	Hoyto et al., 2008b [115]	Murine fibroblasts (L929)	Caspase activity	872 MHz (CW and GSM) 5 W/kg 1 h	No effect	Non-blinded analysis
16	Joubert et al., 2008 [116]	Human neuroblastoma (SH-SY5Y) cells	Morphological hallmarks Caspase activity DNA fragmentation Apoptosis signaling	900 MHz (CW) 2 W/kg 900 MHz (GSM) 0.25 W/kg 24 h	No effect	Non-blinded analysis
17	Joubert et al., 2007 [117]	Primary rat cortical neurons	Morphological hallmarks Caspase activity DNA fragmentation	900 MHz (GSM) 0.25 W/kg 24 h	No effect	Non-blinded analysis
18	Lin et al., 2017 [118]	Mouse Leydig cells	PE	1950 MHz (GSM-talk) 3 W/kg 24 h	No effect	Non-blinded analysis No positive control
19	Liu et al., 2014 [119]	Mouse spermatocyte-derived (GC-2) cells	PE	1800 MHz (GSM) 1, 2, 4 W/kg 24 h (5 min on/10 min off cycles)	No effect	No positive control
20	Merola et al., 2006 [120]	Human neuroblastoma (LAN-5) cells	Caspase activity PARP cleavage	900 MHz (GSM), 1 W/kg 24 to 72 h	No effect	Non-blinded analysis
21	Moquet et al., 2008 [121]	Murine neuroblastoma (N2a) cells	Caspase activity DNA fragmentation PE	935 MHz (CW, GSM- basic, GSM-talk) 2 W/kg 24 h	No effect	-
22	Palumbo et al., 2008 [122]	Human lymphocytes; human lymphoblastoid (Jurkat) cells	Caspase activity PARP cleavage PE	900 MHz (GSM) 1.35 W/kg 1 h	Increase in caspase-3 activity in proliferating but not in resting cells. No effect on PARP cleavage and PE.	Increase in caspase-3 activity not related to apoptosis.
23	Sanchez et al., 2007 [123]	Human skin cells and reconstructed human epidermis	PE	900 MHz (GSM) 2 W/kg 48 h	No effect	Non-blinded analysis
24	Sanchez et al., 2006 [124]	Primary human skin cells	PE	1800 MHz (GSM) 2 W/kg 48 h	No effect	Non-blinded analysis
25	Simon et al., 2013 [125]	Primary human melanocytes and keratinocytes cells	Morphological hallmarks Caspase activity Apoptosis signaling	900 MHz (GSM) 2 W/kg 6 h	No effect	Non-blinded analysis No positive control
26	Terro et al., 2012 [126]	Primary cerebral cortical cells of rat embryos	Morphological hallmarks Caspase activity	900 MHz (GSM) 0.25 W/kg 24 h	No effect	Non-blinded analysis
27	Wang et al., 2015 [127]	Primary murine Bone marrow Mesenchymal stem cells (BM-MSCs)	PE	2.856 GHz (PW) 4 W/kg 6 min	No effect	Non-blinded analysis
28	Zeni et al., 2012 [128]	Rat neuronal cells (PC12)	PE	1950 MHz (UMTS) 10 W/kg 24 h	No effect	-
29	Zhang et al., 2017 [129]	Mouse spermatocyte-derived cells (GC-1)	DNA fragmentation Caspase activity PE	1950 MHz (UMTS) 3 W/kg 24 h	No effect	-
30	Zhou et al., 2019 [130]	Rat pheochromocytoma (PC12) cells	PE	2856 MHz 4 W/kg 8 h/day for 2 days	No effect	Non-blinded analysis No positive control
31	Zielinski et al., 2020 [131]	Murine microglial cells (N9), Human neuroblastoma cells (SH-SY5Y)	PE Apoptosis signaling	935 MHz (GSM) 4 W/kg 2 and 24 h (2 min on/2 min off)	No effect	-

Abbreviations: CDMA: code division multiple access; CW: continuous wave; DTX: discontinuous transmission; FDMA: frequency division multiple access; GSM: global system for mobile communication; iDEN: integrated digital-enhanced network; LTE: long-term evolution; MMP: mitochondrial membrane potential; PARP: Poly (ADP-ribose) polymerase; PE: Phosphatidylserine externalization; PM: pulse modulated; PW: pulsed wave; TDMA: time division multiple access; UMTS: universal mobile telecommunications system.

**Table 4:** Overview of studies that reported statistically significant alteration of the apoptotic process following RF-EMF exposures.

ID	Reference	Cell Type	Biological Endpoint	Exposure Conditions	Results	Comment
1	Borovkova et al., 2017 [132]	C6 rat glial cells	MMP modification	150 GHz 32 W/m^2^ 0 to 5 min	Increase in apoptotic cells over time exposure in exposed samples	Non-blinded analysis No positive control
2	Buttiglione et al., 2007 [133]	Human neuroblastoma cell line SH-SY5Y	DNA fragmentation Apoptosis signaling	900 MHz (GSM) 1 W/kg 5 min, 15 min, 30 min, 6 h, 24 h	Increase in apoptotic sub-G1 DNA content at 24h exposure time, and downregulation of Bcl-2 at 6 and 24 h exposure times	Non-blinded analysis No positive control
3	Canseven et al., 2015 [134]	Burkitt’s lymphoma (Raji) cells	PE	1800 MHz (GSM) 0.35 W/kg 24 h	Increased apoptosis by RF	No positive control
4	Hou et al., 2015 [135]	Mouse embryonic fibroblasts (NIH/3T3)	PE	1800 MHz (GSM talk- mode) 2 W/kg 0.5–8 h (5 min on/30 min off cycles)	Increased apoptosis after 1, 4 and 8 h RF exposure; no effect after 0.5, 2 and 6 h.	Non-blinded analysis No positive control
5	Joubert et al., 2006 [136]	Primary rat cortical neurons	Morphological hallmarks Caspase activity DNA fragmentation	900 MHz (CW) 2 W/kg 24 h	Increased apoptosis (morphological hallmarks and DNA fragmentation) immediately after and 24 h post-RF exposure; no effect on caspase-3 activity; increase in AIF-positive nuclei soon after and 24 h post-exposure.	2 °C increase in RF-exposed cultures. Thermal effects excluded by ad hoc experiments.
6	Li et al., 2020 [137]	Mouse embryonic fibroblasts NIH/3T3	PE Apoptosis signaling	1800 MHz 2 W/kg 12, 24, 36, 48 h (5 min on/10 min off)	Increased apoptosis after 48 h RF exposure; no effect after 12, 24 and 36 h.	Non-blinded analysis No positive control
7	Nikolova et al., 2005 [138]	Mouse neural progenitor stem cells	DNA fragmentation MMP modification Apoptosis signaling	1710 MHz (GSM) 1.5 W/kg 48 h (5 min on/30 min off cycles)	Upregulation of some genes. No effect on other parameters investigated.	No positive control
8	Sefidbakht et al., 2014 [139]	Human embryonic kidney (HEK293T) cells	Caspase activity	940 MHz 0.09 W/kg 15, 30, 45, 60 and 90 min	Increase after 45 and 90 min RF exposure; no effect after 15, 30 and 60 min exposure.	Non-blinded analysis No positive control
9	Yoon et al., 2011 [140]	Human dermal papilla cells	Apoptosis signaling	1763 MHz (CDMA) 10 W/kg 1 h/day for 7 days	Increased expression of Bcl-2 and phosphorylation of MAPK-1.	Non-blinded analysis No positive control
10	Zhao et al., 2020 [141]	A375 Human Melanoma Cells	PE Caspase activity	35.2 GHz 1.6 W/m^2^ 15/30/60/90 min	Increase in apoptosis (PE) at all exposure durations. Upregulation of caspase-3 and caspase-8	Non-blinded analysis No positive control
11	Zuo et al., 2014 [142]	Differentiated rat neuronal cells (PC12)	PE Morphological hallmarks DNA fragmentation MMP modification Apoptosis signaling Caspase activity PARP cleavage	2856 MHz 100–1000 W/m^2^ 5 min	No effect at 10 mW/cm^2^ Increased PE at 30, 50 and 100 mW/cm^2^ at 6h post RF-exposure. At 30 mW/cm^2^ alteration of all the endpoints investigated.	No positive control

Abbreviations: AIF: apoptosis inducing factor; Bcl-2: B-cell lymphoma 2; CDMA: code division multiple access; CW: continuous wave; GSM: global system for mobile communication; MAPK-1: mitogen-activated protein kinase-1; MMP: mitochondrial membrane potential; PARP: Poly (ADP-ribose) polymerase; PE: Phosphatidylserine externalization

## Data Availability

Not applicable.

## Supplementary Materials

The following supporting information can be downloaded at: https://www.mdpi.com/article/10.3390/ijms23042322/s1.
