# Supplementary material for: Radiofrequency Electromagnetic Field Exposure and Apoptosis: A Scoping Review of In Vitro Studies on Mammalian Cells
Source: Int J Mol Sci. 2022 Feb 19;23(4):2322. doi: 10.3390/ijms23042322 (PMC8877695; doi:10.3390/ijms23042322)
Supplement: Supplementary file 1 [file ijms-23-02322-s001.zip › Supplementary Materials/Table S2 - Search queries.docx]

**PubMed search query**

(electromagnetic fields [MeSH Terms] OR microwaves[MeSH Terms] OR radio waves[MeSH Terms] OR radiation, nonionizing[MeSH Terms] OR radiofrequency[Title/Abstract] OR radio frequency[Title/Abstract] OR cellular phone[MeSH Terms] OR mobile phone*[Title/Abstract] OR mobile telephone* [Title/Abstract] OR base station*[Title/Abstract] OR GSM[Title/Abstract] OR UMTS[Title/Abstract] OR mobile communication[Title/Abstract])

AND

(apoptosis OR apoptotic [Title/Abstract] OR programmed cell death [Title/Abstract] OR cell death[Title/Abstract] OR annexin [Title/Abstract] OR tunnel [Title/Abstract] OR phosphatidylserine [Title/Abstract] OR mitochondrial pathway [Title/Abstract] OR death receptor [Title/Abstract] OR chromatin condensation [Title/Abstract] OR caspase [Title/Abstract] OR caspase cleavage [Title/Abstract])

AND

(in vitro [MeSH Terms] OR in vitro [Title/Abstract] OR cell culture*[Title/Abstract] OR cell culture*[MeSH Terms] OR mammalian cells [Title/Abstract] OR mammalian cells [MeSH Terms])

NOT

(UV [Title/Abstract] OR UVA [Title/Abstract] OR UVB [Title/Abstract] OR ultraviolet [Title/Abstract] OR ablation [Title/Abstract] OR 50 Hz [Title/Abstract] OR 60 Hz [Title/Abstract] OR MRI [Title/Abstract] OR low frequency [Title/Abstract])

Filters: Journal Article; English

**Web of Science search query**

TS= (“electromagnetic field*” OR microwaves* OR “radio waves” OR “non-ionizing radiation*” OR radiofrequency OR “radio frequency” OR “radiofrequency radiation” OR “cellular phone*” OR “mobile phone*” OR “mobile telephone*” OR “base station*” OR GSM OR UMTS OR “mobile communication*” OR MHz OR GHz OR “millimeter wave”* OR millimeter-wave OR TeraHertz OR THz)

AND

TS= ("in vitro" OR invitro OR "cell culture*" OR cell* OR "cell line*" OR cellular OR "human peripheral lymphocyte*")

AND

TS= (“apoptosis” OR “apoptotic” OR “programmed cell death” OR “cell death” OR “annexin” OR “tunnel” OR “phosphatidylserine” OR “mitochondrial pathway” OR “death receptor” OR “chromatin condensation” OR “caspase” OR “caspase cleavage”)

NOT

TS = (“static field” OR “Radiofrequency Ablation”)

AND Language: English AND Timespan=All years AND Web of Science Core Collection database AND Article AND Reviews
